# Are carfentanil and acrylfentanyl naloxone resistant?

**DOI:** 10.3389/fpsyt.2024.1359851

**Published:** 2024-02-20

**Authors:** Michael G. Feasel, Theodore S. Moran, Boyle C. Cheng, Saadyah Averick

**Affiliations:** ^1^ Defense Advanced Research Projects Agency (DARPA), Biological Technologies Office (BTO), Arlington, VA, United States; ^2^ U.S. Army DEVCOM Chemical and Biological Center, Aberdeen, MD, United States; ^3^ Neuroscience Institute, Allegheny Health Network, Pittsburgh, PA, United States

**Keywords:** opioid, fentanyl, overdose, naloxone, *in vitro*

## Abstract

The rapid rise in deaths since 2012 due to opioid poisoning is correlated with the proliferation of potent synthetic opioid agonists such as fentanyl, acrylfentanyl, and carfentanil. The efficacy of frontline antidotes such as naloxone in reversing such poisoning events has been questioned, and the possibility of naloxone-resistant synthetic opioids has been raised. In this manuscript, we applied *in vitro* techniques to establish the median effective inhibitory concentrations for fentanyl, acrylfentanyl, and carfentanil and subsequently evaluate naloxone’s ability to reverse agonist–receptor interactions.

## Introduction

Synthetic mu-opioid receptor (MOR) agonists derived from the 4-anilinopiperidine scaffold are responsible for the exponential increase in opioid overdose deaths and poisoning (1–7). In the past several years, progress toward stemming the tide of death has been reduced due to the increasing potency of synthetic opioids and limited healthcare access (8–10). These compounds are relatively straightforward to prepare from available precursors and are used either as adulterants to other opioids or directly used (11–14). Multiple factors that contribute to the lethality of synthetic opioids are their a) rapid onset, b) high potencies (20 to 2000 times greater than morphine), and c) relatively long pharmacokinetics compared to frontline antidotes (15–21). These factors have led to critical questioning of the potency and efficacy of the frontline antidote naloxone to reverse synthetic opioid poisoning (22–26).

Two major mechanisms postulating the limitations of standard naloxone antidote dosing, in the event of synthetic opioid poisoning, have been put forward. The *renarcotization theory* states that the pharmacokinetic mismatch between naloxone and synthetic opioids causes a single naloxone dose to be insufficient at sustained overdose reversal (27–33). *Naloxone resistance* is a theorized phenomenon where naloxone has a limited and dulled capacity to reverse synthetic opioid agonist poisoning (22, 34). The idea of naloxone resistance was first put forward by Maryanoff and coworkers in 1982 and has since found reference in both popular press and scientific literature (35). The competing theory of renarcotization being the source of naloxone’s limitations stems from the metabolic mismatch between naloxone and synthetic opioids (36–42). Naloxone is, compared to synthetic opioids, relatively hydrophilic and is rapidly metabolized by the UGT2B7 glucuronidase enzyme leading to the production of the brain-impenetrable naloxone-3-glucuronide (43, 44). In contrast, synthetic opioids are passively absorbed by adipose tissue due to inherent hydrophobicity and, upon naloxone’s metabolism, are able to agonize the MOR, resulting in renarcotization (17, 45–48). In this manuscript, we sought to evaluate two potent synthetic MOR agonists both postulated to have potential naloxone resistance, resulting in the need for new countermeasures and antidotes for these compounds.

**Figure 1:**
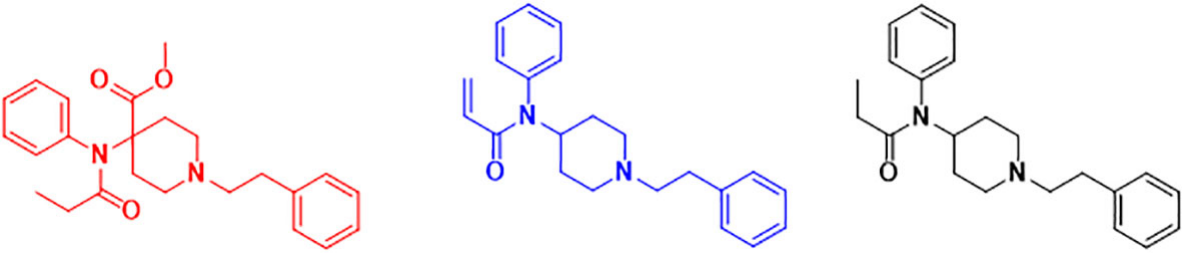
Chemical structures of fentanyl (black), acrylfentanyl (blue), and carfentanil (red).

To achieve our aims, an *in vitro* method using a commercially validated CHO-K1 cell line expressing human MOR was employed to directly determine a median effective concentration (EC_50_) for fentanyl, carfentanil, and acrylfentanyl (**Figure 1**). Median inhibitory concentrations (IC_50_) for fentanyl, carfentanil, and acrylfentanyl after competition with naloxone were also generated to assess the reversibility of the receptor–ligand interaction.

## Materials and methods

### Chemicals

The LANCE Ultra cAMP assay point kit and 384-well ProxiPlate were purchased from Perkin Elmer (Shelton, CT, USA). The LANCE Ultra kit consisted of cAMP Standard (50 µM), Eu-cAMP tracer (ULight™-anti-cAMP), cAMP Detection Buffer, and BSA Stabilizer. Carfentanil was synthesized at the U.S. Army DEVCOM Chemical Biological Center (CBC) (APG, MD, USA), and purity was verified by ^13^C and ^1^H NMR. [d-Ala^2^,NMe-Phe^4^,Gly-ol^5^]-Enkephalin (DAMGO) was purchased from Tocris Bioscience (Park Ellisville, MO, USA). Hanks’ Balanced Salt Solution (HBSS) 1X, 4-(2-hydroxyethyl)-1-piperazineethanesulfonic acid (HEPES) 1M, Versene Solution, and Geneticin were procured from Life Technologies (Grand Island, NY, USA). Dimethyl sulfoxide (DMSO), 3-isobutyl-1-methylxanthine (IBMX), and forskolin were procured from Sigma-Aldrich (St. Louis, MO, USA). Dulbecco’s Phosphate-Buffered Saline (DPBS)/Modified Buffer and Ham’s F-12 Media were procured from HyClone Laboratories, Inc. (Logan, UT, USA). Fetal bovine serum (FBS) was procured from Mediatech, Inc. (Manassas, VA, USA). Fentanyl citrate was procured from Mallinckrodt Pharmaceuticals (St. Louis, MO, USA). Acrylfentanyl HCl was procured from Cayman Chemical (Ann Arbor, MI, USA).

### Cell line

ValiScreen CHO-K1 cells expressing human MOR (ES-542-C) were purchased from Perkin Elmer, Inc. (Waltham, MA, USA). The cells were kept frozen in liquid nitrogen storage (vapor phase) until they were cultured. The cells were grown in accordance with product literature provided by Perkin Elmer. The cell cultures were split when they reached ~60%–80% confluence, and no cells were used past passage 10. Cells were used for opioid assay when they met the requirements described in the product literature (i.e., 60%–80% confluence). Before use, cellular solutions used in plating were counted on a Vi-CELL XR hemocytometer (Beckman Coulter Life Sciences, Indianapolis, IN, USA). The cells were plated at a concentration of 2.0 × 10^5^ cells/mL.

### Incubation and standard solutions

Standard solutions of fentanyl, carfentanil, acrylfentanyl, and naloxone (10 mM) were made in DMSO and stored until use in a freezer at −20°C. A standard solution of DAMGO (1.95 mM) was made in sterile water. Working solutions of fentanyl, carfentanil, acrylfentanyl, naloxone, and DAMGO (500 µM) were prepared immediately before the assay was performed in a fresh stimulation buffer. Stimulation buffer, forskolin dilutions, and cAMP standards were made, as needed, in accordance with the Lance Ultra cAMP assay protocol immediately before the assay was performed.

For the competition assay to assess naloxone requirements, each agonist’s EC_90_ was co-incubated in each well concurrently with naloxone concentrations ranging from 100 µM to 10 fM in full log intervals. A zero-naloxone concentration control was also included.

### Statistical analysis

Median effective concentrations of DAMGO, fentanyl, carfentanil, and acrylfentanyl were calculated using GraphPad Prism v9.0.1 (GraphPad Software, Inc., La Jolla, CA, USA). Data were first normalized to the minimal and maximal responses of the control compound, DAMGO, and then non-linear regression was fit to each dose–response profile using the three-parameter [agonist] vs. normalized response function within the software. Ordinary one-way ANOVA tests using multiple comparisons were then conducted to assess the statistical significance of the EC_50_ values. EC_90_ values were then calculated similarly for each of the agonists for use in the competition assay that followed.

Median inhibitory concentrations of naloxone were then calculated for challenges with the EC_90_ values of DAMGO, fentanyl, carfentanil, and acrylfentanyl. This was performed similarly to the EC_50_ determination study with the exception that for this set of experiments, the non-linear regression function used was a three-parameter [inhibitor] vs. normalized response. Ordinary one-way ANOVA tests using multiple comparisons were similarly conducted to assess the statistical significance of the IC_50_ values against each of their challenge doses.

## Results and discussion

### Agonist dose–response

**Figure 2:**
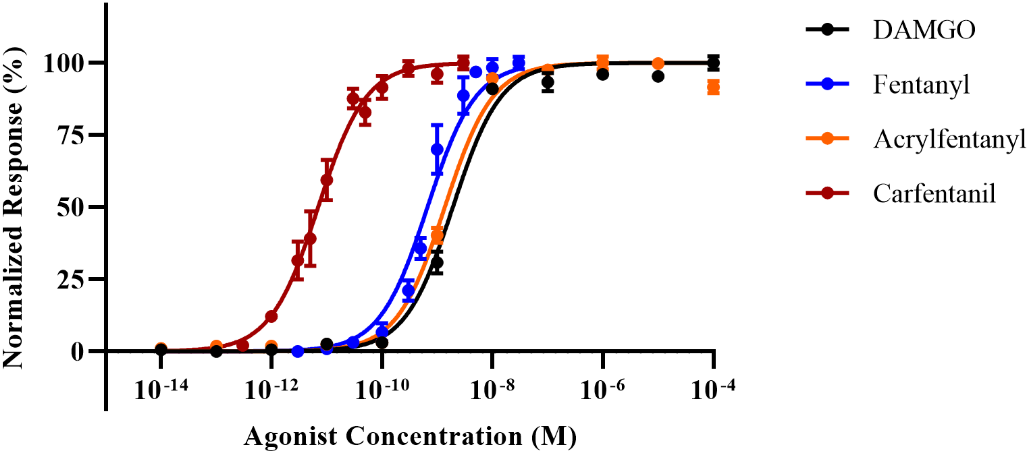
Dose–response curves for [d-Ala^2^,NMe-Phe^4^,Gly-ol^5^]-enkephalin (DAMGO), fentanyl, carfentanil, and acrylfentanyl. Data points plotted as mean ± standard error of the mean (SEM).

To establish baseline agonist–receptor activation, we generated full dose–response curves for DAMGO, fentanyl, carfentanil, and acrylfentanyl, and we determined EC_50_, EC_90_, and relative potency to fentanyl (**Figure 2**). By using a functional cAMP response cell model to determine agonist potential, we are able to directly ascertain drug potency as compared to pure receptor-radio-ligand binding assays, which may not reflect the true potency of agonists. Our studies were able to determine the compounds’ relative potency to fentanyl (**Table 1**) and several interesting points based on the EC_50_ values. Contrary to the initial literature report by Maryanoff, which found acrylfentanyl to have a potency greater than that of fentanyl, we found that acrylfentanyl had only ~50% of the potency of fentanyl. In contrast, we determined that carfentanil’s potency was nearly 100× that of fentanyl. The determined EC_50_ and EC_90_ values enable naloxone reversal assays.

**Table 1:** Potency values for opioid agonists.

Drug	EC_50_ (nM)	95% CI (nM)	Relative potency (× fentanyl)	*p-*Value	EC_90_ (nM)
DAMGO*	1.92	1.61–2.29	0.35	<0.0001	17.3
Fentanyl	0.667	0.551–0.808	1.0	NA	6.00
Carfentanil*	0.00699	0.00583–0.00837	95	0.0003	0.0629
Acrylfentanyl*	1.37	1.16–1.63	0.49	0.0001	12.3

DAMGO, [d-Ala^2^,NMe-Phe^4^,Gly-ol^5^]-enkephalin.

^*^Statistical significance by one-way ANOVA when compared to EC_50_ of fentanyl.

NA = Not applicable.

### Antagonist performance of naloxone

**Figure 3:**
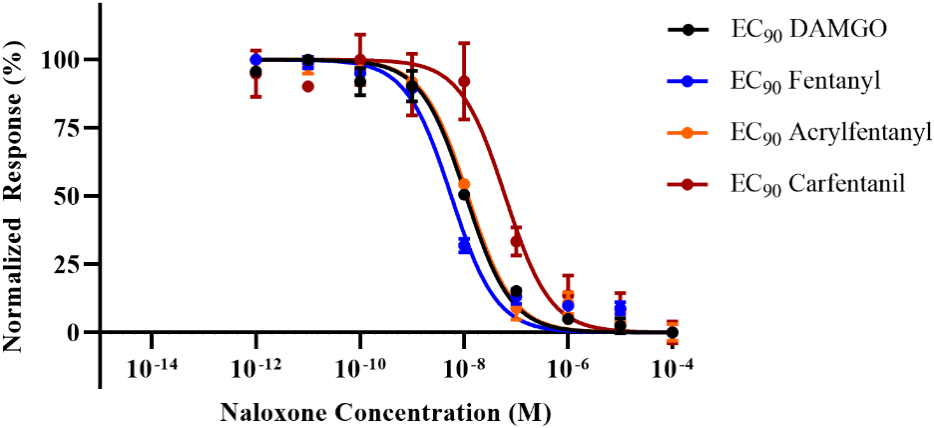
Inhibition dose–response curves for naloxone against challenges of the EC_90_ for each agonist. Data points plotted as mean ± SEM.

In order to directly ascertain naloxone’s ability to reverse the agonist activity of the synthetic opioids, cells were stimulated with an EC_90_ of each compound, and a dose–response curve with naloxone was generated against each compound. The EC_50_ and EC_90_ values (**Table 1**) were determined in the agonist assay for fentanyl and acrylfentanyl. Cells were incubated with EC_90_ of DAMGO, fentanyl, carfentanil, or acrylfentanyl and log-dosed concentrations of naloxone. The experimental design allowed for the determination of the concentration of naloxone (antagonist) required to reverse an EC_90_ dose of a synthetic opioid (agonist). By maintaining an EC_90_ of agonist in each well and varying the concertation of naloxone, backward-S dose–response curves (**Figure 3**) were generated, and the IC_50_ values could be calculated for naloxone against the respective synthetic opioids (**Table 2**).

**Table 2:** IC_50_ values for naloxone when challenged with EC_90_ values of DAMGO, fentanyl, and acrylfentanyl.

Drug	IC_50_ (nM)	95% CI (nM)	Relative naloxone requirement (× fentanyl)	*p-*Value
DAMGO	10.8	8.40–14.0	1.9	0.9766
Fentanyl	5.64	4.29–7.41	1.0	NA
Carfentanil*	61.4	33.9–110.3	10.9	0.0002
Acrylfentanyl	11.8	9.26–15.0	2.09	0.9623

DAMGO, [d-Ala^2^,NMe-Phe^4^,Gly-ol^5^]-enkephalin.

^*^Statistical significance by one-way ANOVA when compared to the naloxone IC_50_ against fentanyl.

NA = Not applicable.

While the results of the agonist reversal assay clearly demonstrate that the agonist activities of acrylfentanyl and carfentanil can both be reversed with naloxone, several interesting phenomena were observed. The first observation was that although acrylfentanyl had approximately one-half the potency of fentanyl, this compound required nearly double the amount of naloxone to reverse its agonist activity vs. fentanyl. Carfentanil, however, demonstrated potency roughly 100× that of fentanyl and required a significantly greater concentration of naloxone in order to antagonize a challenge of its EC_90_. Further, this demonstrates that reports of naloxone resistance need to be verified scientifically, as carfentanil appears to require more naloxone in order to reverse its effects, but this is not due to any detection of irreversible binding, but rather in this test, the system appears to be due to affinity for the receptor and functional agonistic potency.

While this study aims to equivocally characterize the receptor–ligand interaction of fentanyl-class opioid agonists at the MOR, it does not provide guidance for dosing requirements in instances of treatment of opioid overdose. This study merely aims to provide evidence that the interaction between a ligand, acrylfentanyl, and its primary target receptor is indeed a reversible interaction and thus able to be reversed in proper conditions, contrary to what has been reported in mass media. In agreement with our approach and conclusions, studies performed in humans with radiolabeled carfentanil also demonstrate rapid distribution of naloxone to the brain and subsequent displacement of [^11^C]-carfentanil shortly after administration followed by rapid redistribution of naloxone out of the central nervous system (CNS). The lower receptor affinity of naloxone enables its displacement from the MOR by carfentanil, practically demonstrating how naloxone resistance may occur in living systems (40). These data support our hypothesis that carfentanil, despite its heroic potency at the MOR, is able to be displaced even by nominal dosing of naloxone. Similarly, the authors of this study acknowledge that their work, too, does not dictate dosing requirements of naloxone or any other opioid antagonist based on the amount of agonist administered; their work merely demonstrates that the agonist is not irreversibly bound to the receptor but happens to be an incredibly potent agonist, functionally, although it is fully reversible at the molecular level.

## Conclusions

In this study, we applied an *in vitro* live cell receptor activation assay method to determine EC_50_ values for synthetic MOR agonists. These values were then used to determine the amount of naloxone required to inhibit agonist activity. This straightforward approach allowed for the direct determination that naloxone is capable of reversing the agonist effects of even the most potent of known synthetic opioids (carfentanil). Nevertheless, our data also indicate that the amount of naloxone needed to reverse the agonist activity of synthetic opioids varies compared to fentanyl. Our data show that naloxone is an effective inhibitor of synthetic opioids. Our future studies aim to translate these results to an *in vivo* animal model of synthetic opioid poisoning to determine the amount of naloxone needed to successfully reverse fentanyl toxicity vs. acrylfentanyl and carfentanil. Finally, this study highlights the need for a longer-acting MOR antagonist that can be sufficiently dosed at levels that do not induce precipitated opioid withdrawal yet can still reverse the most potent synthetic opioids.

## Data availability statement

The original contributions presented in the study are included in the article/supplementary material. Further inquiries can be directed to the corresponding author.

## Author contributions

MF: Investigation, Methodology, Writing – original draft, Writing – review & editing, Conceptualization, Data curation, Formal analysis, Project administration, Resources, Software, Supervision, Validation, Visualization. TM: Data curation, Formal analysis, Investigation, Writing – original draft. BC: Writing – original draft, Writing – review & editing. SA: Writing – original draft, Writing – review & editing, Funding acquisition, Investigation, Methodology.
